# Optimal Management of Bleeding and Thrombosis in Total Knee Arthroplasty: Tranexamic Acid With Acetylsalicylic Acid or Low-Molecular-Weight Heparin

**DOI:** 10.7759/cureus.83154

**Published:** 2025-04-28

**Authors:** Zafer Uzunay, Ahmet Burak Satilmis, Tolgahan Cengiz, Muhammed Nadir Yalcin

**Affiliations:** 1 Orthopaedics and Traumatology, Medicalpark Adana Hospital, Adana, TUR; 2 Orthopaedics and Traumatology, Taşköprü State Hospital, Kastamonu, TUR; 3 Orthopaedics and Traumatology, Faculty of Medicine, Ondokuz Mayıs University, Samsun, TUR; 4 Orthopaedics and Traumatology, Karabük Medikar Hospital, Karabük, TUR

**Keywords:** acetylsalicylic acid, low-molecular weight heparin, perioperative tranexamic acid, total knee arthroplasty, venous thromboembolism (vte)

## Abstract

Objective

This study compared the efficacy and safety of combining tranexamic acid, which reduces bleeding, with acetylsalicylic acid (ASA) or low-molecular-weight heparin (LMWH), which is used for venous thromboembolism (VTE) prophylaxis in patients undergoing total knee arthroplasty (TKA).

Materials and methods

A retrospective analysis was conducted on 86 patients who underwent primary unilateral TKA between 2014 and 2020. Patients were divided into Group 1 (intravenous tranexamic acid (TA) + acetylsalicylic acid) and Group 2 (intravenous tranexamic acid + low-molecular-weight heparin). Demographic data, perioperative blood loss, transfusion requirements, and complications were recorded and analyzed.

Results

Group 1 showed significantly lower total blood loss (TBL), drainage volume, and transfusion rates than Group 2. No significant differences were observed in hidden blood loss (HBL) or maximum hemoglobin drop between the groups. Postoperative complications, including VTE, wound issues, and systemic adverse events, were comparable between the two groups. The length of hospital stay was shorter in Group 1.

Conclusion

The combination of TA with ASA demonstrated superior outcomes in blood conservation and transfusion rates compared to TA with LMWH, with no increase in postoperative complications. These findings suggest that ASA is a safe and effective option for VTE prophylaxis in TKA patients when used with TA.

## Introduction

Knee osteoarthritis is the most common cause of knee pain and disability in older people [1]. Total knee arthroplasty (TKA) is an effective treatment for patients with advanced degenerative knee osteoarthritis. Primary total knee arthroplasty surgery is projected to increase by 673% to 3.48 million surgeries in the United States from 2005 to 2030 [2]. As with any surgery, TKA surgery also has complications. Bleeding and venous thromboembolism (VTE) are common complications of TKA [3].

In recent years, the risk of developing acute postoperative anemia and the need for allogeneic blood transfusion have increased after TKA surgeries, which have become routine orthopedic procedures and have been performed quite frequently [4]. Therefore, orthopedic surgeons have developed various protocols in recent years to reduce the amount of bleeding and the need for transfusion. Tranexamic acid (TA), an antifibrinolytic agent used for this purpose, significantly reduces the need for transfusion and has fundamentally changed the management of bleeding in arthroplasty surgery. It is now routinely used in TKA surgery [5, 6].

Prophylaxis is recommended in TKA patients because of the relatively high risk of venous thromboembolism (VTE). The recently revised American College of Chest Physicians (ACCP) and American Academy of Orthopaedic Surgeons (AAOS) guidelines recommend mechanical and chemical prophylaxis. Although different chemical agents can be used according to these guidelines, they differ in effectiveness and bleeding risks [7]. The ideal prophylaxis regimen is still controversial due to complications of thromboprophylaxis agents, such as bleeding, prolonged wound drainage, and infection. Therefore, while minimizing the risk of VTE, unwanted and sometimes fatal bleeding complications should be avoided [8]. Our clinic routinely uses acetylsalicylic acid (ASA) or low-molecular-weight heparin (LMWH) for VTE prophylaxis.

Pharmacological agents commonly used to inhibit platelet aggregation and prevent intravascular clot formation include acetylsalicylic acid (ASA) and low-molecular-weight heparin (LMWH). This study aimed to compare the efficacy of these two agents in combination with tranexamic acid to control bleeding to reduce the risk of venous thromboembolism (VTE) after thromboprophylaxis and to evaluate the possible complications of these combinations. No study in the literature compares the efficacy and safety profiles of tranexamic acid and ASA or tranexamic acid and LMWH combinations. Therefore, this study's results may contribute to managing bleeding risk and optimizing prophylactic treatment strategies in clinical practice.

## Materials and methods

Data from patients who underwent TKA between June 2014 and December 2020 in a tertiary university hospital's Orthopedics and Traumatology Clinic were analyzed retrospectively. The patients were evaluated in two groups to compare TA, an antifibrinolytic drug routinely used to reduce bleeding in TKA surgeries, with the two most commonly used agents for VTE thromboprophylaxis (ASA-LMWH). Patients who underwent IV (intravenous) TA and used 100 mg acetylsalicylic acid in the postoperative period were named as Group 1, and patients who underwent IV-TA and used 4000 anti-Xa IU/0.4 ml enoxaparin sodium in the postoperative period were named as Group 2.

Patients aged between 50-85 years who underwent unilateral primary total knee arthroplasty with anterior skin incision and quadriceps-preserving medial parapatellar approach were included in the study. The files of 283 patients were reviewed. Exclusion criteria were determined as patients who underwent knee arthroplasty due to trauma or tumor, patients who underwent bilateral TKA, patients who underwent revision TKA, patients who had previously undergone any knee surgery, those allergic to tranexamic acid, acetylsalicylic acid and low-molecular-weight heparin, those with bleeding disorders, patients using warfarin or heparin, patients with Paget disease, those with a history of deep vein thrombosis or pulmonary embolism, patients with renal failure with creatinine levels >250 micromol/l, pregnant women, patients who did not have regular outpatient clinic follow-up in the 6 months after surgery, and those with missing or missing data. Considering these criteria, 53 patients were included in the study in Group 1 and 33 in Group 2. As this was a retrospective study, the choice between low-molecular-weight heparin (LMWH) and acetylsalicylic acid (ASA) was determined by institutional protocols at the time and the attending physician’s clinical judgment, taking into account patient comorbidities, bleeding risk, and contraindications.

All patients were operated on under general or regional anesthesia, using a tourniquet and the same surgical technique. The same design implant was used in all patients, and drains were applied to all patients after surgery. IV-TA (Transamine, Actavis, Dublin, Ireland) was administered before surgery: 1 g TA in 150 ml 0.9% sodium chloride solution for 30 minutes. After surgery, 250 mg TA was administered in 150 ml 0.9% sodium chloride solution in four doses at 6-hour intervals. The patients' demographic characteristics and preoperative medical histories were recorded by reviewing their files. The duration of hospitalization was taken as the period from the surgery date to discharge. Information on the type of anesthesia (general/spinal) and the American Society of Anesthesiologists (ASA) score was obtained from the anesthesia records. Since all cases included in the study were operated on under a tourniquet, intraoperative blood loss was negligible. Operation times were recorded.

In this study, all patients were monitored for venous thromboembolism (VTE), including deep vein thrombosis (DVT) and pulmonary embolism (PE), during their hospital stay and throughout the postoperative period. Clinical evaluation for signs of VTE was routinely performed during hospitalization and at follow-up visits scheduled within six months postoperatively. In cases of clinical suspicion, diagnostic confirmation was obtained using Doppler ultrasonography for DVT and computed tomography pulmonary angiography for PE. The absence of symptomatic VTE during this follow-up period was accepted as an indicator of thromboprophylaxis success. Routine blood tests, including hemoglobin (Hb), hematocrit (Hct), platelets (PLT), and coagulation function (activated partial thromboplastin time (aPTT), prothrombin time (PT), and hepatic and renal measurements were reviewed from the records during the preoperative preparation period. Blood loss was calculated using the blood volume calculation formula defined by Nadler and the total blood loss formula defined by Gross. Hidden blood loss (HBL) was calculated by subtracting the blood volume coming from the drain from the total blood loss (intraoperative blood loss (IBL) was ignored because the study was conducted under a tourniquet). Other values ​​examined in the study were maximum Hb decrease, total blood loss (TBL), blood volume coming from the drain, transfusion amount, and hospitalization time. The maximum Hb decrease was found by subtracting the lowest Hb value before or after blood transfusion from the preoperative Hb value. During hospitalization, postoperative complications, including acute renal and hepatic failure, wound complications (prolonged drainage, infection, delayed wound healing), and clinically insignificant bleeding (unexpected hematoma, bleeding, and ecchymosis), were recorded.

The study was conducted with approval from the Clinical Research Ethics Committee of Karabuk University Training and Research Hospital (Number: 2020/377 Date: 10.11.2020).

Statistical analysis

Data were analyzed using IBM SPSS V26 (Armonk, NY: IBM Corp). The Shapiro-Wilk test was used to examine compliance with normal distribution. Chi-square and Fisher’s Exact tests were used to compare categorical variables according to groups. The independent two-sample t-test was used for normally distributed data, and the Mann-Whitney U test was used for non-normally distributed data to compare quantitative variables according to binary groups. Categorical measurements were summarized as numbers and percentages, and numerical measurements were summarized as mean ± standard deviation (median and minimum-maximum where necessary). Statistical significance level was taken as 0.05 in all tests.

## Results

A total of 86 patients were included in the study. There were 53 patients in Group 1 and 33 patients in Group 2. The mean age of Group 1 was 66.28 ± 6.40 years, and the mean age of Group 2 was 68.18 ± 5.57 years. When gender distribution was considered in our study, 41 females and 12 males were in Group 1, and 27 females and six males were in Group 2. BMI was 30.45 ± 5 kg/m^2^ in Group 1 and 33.12 ± 6.67 kg/m^2^ in Group 2 (Table 1). In our study, 26 of the patients in Group 1 had surgery on their right knees, 27 on their left knees, and 15 in Group 2 had surgery on their right knees and 18 on their left knees. When preoperative hemoglobin was examined, it was found to be 13.57±1.43 g/dL in Group 1 and 13.18±1.33 g/dL in Group 2. When preoperative hematocrit values ​​were examined, it was found to be 41.30±3.69% in Group 1 and 40.88±4.03% in Group 2. Blood volume values ​​were 447.79±63.67 ml in Group 1 and 471.10±55.86 ml in Group 2.In the comparisons made, no statistically significant difference was observed between preoperative Hb, preoperative Hct, and blood volume values ​​(p = 0.214, p = 0.623, and p = 0.88, respectively) (Table 1). Operation time was 92.15 ± 21.752 minutes in Group 1 and 87.24 ± 19.967 minutes in Group 2. No statistically significant difference was found between the groups regarding operation time (p = 0.297).

**Table 1:** Demographic characteristics of patients Hb: Hemoglobin, Hct: Hematocrit, BMI: Body Mass Index

	Group 1 (n=53)	Group 2 (n=33)	p-value
Age (years), mean±SD	66.28 ± 6.404	68.18 ± 5.570	0.164
Gender (male/female), n	12/41	6/27	0.621
Side (Right/Left), n	26/27	15/18	0.745
BMI (kg/m^2^), mean±SD	30.45 ± 5	33.12 ± 6.67	0.038
Preoperative Hb (g/dl), mean±SD	13.57±1.43	13.18±1.33	0.214
Preoperative Hct (%), mean±SD	41.30±3.69	40.88±4.03	0.623
Blood Volume (ml), mean±SD	447.79±63.67	471.10±55.86	0.88

Our study observed a statistically significant difference between the groups regarding ASA score distribution (p = 0.032). No patient with an ASA score of 1 was observed in Group 2 (Table 2). The patients were operated on under spinal or general anesthesia. No statistically significant difference was observed between the distributions of the groups in terms of the type of anesthesia applied (p = 0.367). When the groups were examined in terms of comorbidity, a statistically significant difference was observed between the groups in terms of cardiovascular disease (CVD) and chronic obstructive pulmonary disease (COPD), while no statistically significant difference was observed in terms of diabetes mellitus (DM) and hypertension (HT). (p < 0.001 and p = 0.01, respectively). While no statistically significant difference was observed between the two groups in terms of HBL values, a statistically significant difference was observed between the two groups in terms of TBL values ​​(p < 0.05). Statistically, significantly higher bleeding values ​​were observed in Group 2, where enoxaparin sodium was used (Table 3). A statistically significant difference was observed between the two groups regarding the amount of bleeding from the drain. While the mean drainage was found to be 275 (min. 25 - max. 850) ml in Group 1, it was statistically significantly higher in Group 2 with 450 (min. 50 - max. 850) ml (p = 0.006) (Table 3). When the length of hospitalization was evaluated, a statistically significant difference was observed between the two groups (p = 0.016). Patients in Group 2 had longer hospitalization lengths (Table 3). When the necessity of blood transfusion was evaluated, a statistically significant difference was found between the two groups (p = 0.024). The blood transfusion rate was higher in Group 2 patients who used enoxaparin sodium (Table 4).

**Table 2:** ASA (American Society of Anesthesiologists) Score distribution of patients, n (%)

ASA Score ->	1	2	3	Total	p-value
Group 1	5 (9.4%)	37 (69.8%)	11 (20.8%)	53 (61.6%)	0.032
Group 2	-	19 (57.6%)	14 (42.4%)	33 (38.4%)	
Total	5 (5.8%)	56 (65.1%)	25 (29.1%)	86 (100%)	

**Table 3:** Postoperative data HBL: Hidden blood loss, TBL: Total blood loss

	Group 1	Group 2	p-value
HBL (ml), mean±SD	879.087±393.896	1027.794±410.086	0.097
Drainage (ml)	275 (min. 25 - max. 850)	450 (min. 50 - max. 850)	0.006
TBL (ml)	1133 (min. 148 - max. 2816)	1377 (min. 708 - max. 2486)	0.011
Hospitalization period (days)	4 (min. 3 - max. 10)	5 (min. 3 - max. 9)	0.016

**Table 4:** Patients' need for blood transfusion due to surgery, n (%)

		Group 1	Group 2	Total	p-value
Blood transfusion	(+)	2 (3.8%)	7 (21.2%)	9 (10.5%)	0.024
	(-)	51 (96.2%)	26 (78.8%)	77 (89.5%)	
Total		53 (61.6%)	33 (38.4%)	86 (100%)	

When maximum Hb change values ​​were evaluated, no statistically significant difference was observed between the two groups(p = 0.679) (Table 5). Among the complications, no significant difference was observed between the two groups in comparing prolonged wound drainage complications (p>0.05). In Group 1, one patient had gastrointestinal (GI) bleeding (after discharge), one patient had acute renal failure, and one patient had hyponatremia. When the frequency of complications was evaluated, no difference was observed between the groups (p = 0.523). DVT was observed in two patients in Group 1 and Group 2. PE was not detected in either group. No statistically significant difference was observed between the groups regarding VTE (p = 0.673). These results show that the tranexamic acid + ASA combination causes less bleeding and complications (and lower blood transfusion rates) than the tranexamic acid + LMWH combination.

**Table 5:** Hb (hemoglobin) decrease values ​​in patients after surgery

	Max. Hb decrease (g/dl)
Group 1	3.20
Group 2	3.29
p-value	0.679

## Discussion

The effectiveness of TA, an antifibrinolytic agent, in reducing blood loss and transfusion rates has been reported in many studies, but the ideal administration route is still controversial [9]. The most common methods of TA administration are IV and topical (intra-articular) injection. Some publications suggest that combining the two may have a more positive effect on reducing bleeding than using only one method [10]. We planned this study assuming that TA and anticoagulant drugs may interact with anticoagulant medications and alter their effectiveness when administered systemically. The thromboprophylaxis regimen defined in current guidelines (AAOS, ACCP, and National Institute for Health and Care Excellence (NICE)) is as follows: acetylsalicylic acid is recommended to be administered 6-8 hours after surgery (there is no consensus in the literature regarding the dose and frequency of administration) [11] and enoxaparin sodium is recommended to be administered 12 hours after surgery [12]. The time of administration of anticoagulants to the patient is longer than the half-life of TA; that is, when a single dose IV-TA regimen is applied, TA and anticoagulant agents will not be present in the systemic circulation simultaneously. From this perspective, we thought that a single-dose IV-TA regimen would create limitations in evaluating the interaction with anticoagulants. Therefore, we included patients who received multiple IV-TA regimens when designing the study.

The main aim of our study was to compare the efficacy and safety of acetylsalicylic acid and enoxaparin sodium used for VTE prophylaxis in TKA patients undergoing antifibrinolysis with TA. Balk et al., in their systematic review including five direct comparative studies, compared acetylsalicylic acid and LMWH in terms of PE, symptomatic DVT, and significant bleeding and found no difference in clinical efficacy or safety between acetylsalicylic acid and LMWH [13]. Kapoor et al., on the other hand, published a meta-analysis of 94 randomized controlled clinical trials in which they recorded VTE and bleeding data; they reported no difference in clinical efficacy or safety between acetylsalicylic acid and LMWH (administered once daily) [14]. Wilson et al. reported no significant difference in comparative clinical efficacy between acetylsalicylic acid and LMWH in patients undergoing THA (total hip arthroplasty) or TKA regarding VTE and wound complications [15]. TA, which has significant effects on bleeding and blood loss, was not evaluated when all these studies were examined. We designed our study based on this limitation in the literature. We determined no difference in the efficacy and reliability of the two most common agents used for VTE prophylaxis, acetylsalicylic acid and enoxaparin sodium, in TKA patients who received a standardized antifibrinolytic treatment with TA. Our study's data showed that using acetylsalicylic acid or enoxaparin sodium would not lead to different postoperative complications after the antifibrinolytic effect was achieved with TA.

In our study, we investigated the effects of the most commonly used agents for VTE thromboprophylaxis, acetylsalicylic acid or enoxaparin sodium, on blood loss (HBL, TBL, drainage, maximum Hb decrease) in TKAs receiving multiple IV-TA regimens. We concluded that the use of enoxaparin sodium or acetylsalicylic acid together with multiple IV-TA regimens did not change HBL, but the use of acetylsalicylic acid significantly reduced TBL and drainage compared to enoxaparin sodium. Zou et al. evaluated the effects of acetylsalicylic acid, rivaroxaban, and LMWH on the incidence of DVT, occult blood loss, and wound complications in their prospective randomized controlled study on 324 patients undergoing TKA. In their study, they reported that there was no significant difference in HBL between the group receiving LMWH and the group receiving acetylsalicylic acid [16]. Although this result is consistent with our study, Zou et al. did not share data on TA use in their research, and the study only compared thromboprophylaxis agents with each other. Although many studies make this comparison, it is still being determined how TA, which has been proven effective on bleeding and blood loss and has entered standard use, is effective in these comparisons and with different agents. In this respect, our study will contribute to the existing literature.

In our study, the blood transfusion rate was higher in Group 2 patients who received IV-TA + enoxaparin sodium. In a retrospective cohort analysis of 1244 patients (366 acetylsalicylic acids, 438 enoxaparin, and 440 rivaroxaban) who underwent TKA and THA, Lindquist et al. found the transfusion rates to be 5.2% (19/366) in the acetylsalicylic acid group, 36.1% (158/438) in the enoxaparin group and 25.2% (111/440) in the rivaroxaban group. The transfusion rates of the enoxaparin and rivaroxaban groups were statistically higher than those of the acetylsalicylic acid group [17]. Bala et al. included 1016 patients using acetylsalicylic acid, 6096 patients using enoxaparin, 6096 patients using warfarin, and 5080 patients using factor Xa inhibitors in their retrospective study, and the group receiving acetylsalicylic acid had the lowest transfusion rate, with a blood transfusion rate of 7% [18]. Our results were similar to the findings in the current literature. The blood transfusion rates in both studies were higher than in our study. This is because the studies by Bala et al. and Lindquist et al. compared only different thromboprophylaxis agents without TA. Indeed, TA has been shown to reduce the transfusion rate in TKA in many studies in the literature [19-21].

Matharu et al. included 13 randomized controlled trials in their meta-analysis comparing the efficacy and safety of acetylsalicylic acid with other anticoagulants in patients undergoing arthroplasty. In this study of 6060 patients, it was observed that the risk of side effects such as significant bleeding, wound hematoma, and wound infection in patients receiving acetylsalicylic acid was not statistically significant compared to other anticoagulants (heparin, enoxaparin, dabigatran, rivaroxaban, and warfarin) [22]. Similarly, in our study, postoperative complications were found to be 11.3% (6/53) in Group 1 patients receiving IV-TA + acetylsalicylic acid and 9.1% (3/33) in Group 2 patients receiving IV-TA + enoxaparin sodium, with no significant difference. Our results are consistent with the literature if only our anticoagulant preference is considered. However, there is no comparison between complications under the conditions in which TA is used. In this respect, the results of our study will contribute to the literature. Gutowski et al. reported that using acetylsalicylic acid compared to warfarin following arthroplasty reduced the cost of VTE by shortening the length of hospital stay and decreasing the incidence of PE [23]. Acetylsalicylic acid has anti-inflammatory properties that may aid in restoring and returning functional functions. Keays et al. compared the effects of acetylsalicylic acid and LMWH on the early return of motion of the knee after TKA and found that the acetylsalicylic acid group had a faster return of motion [24]. When the length of stay was evaluated, Group 1, which received IV-TA + acetylsalicylic acid, had a statistically significant shorter length of stay.

The most important strength of our study is that it fills a gap in the literature by comparing ASA and LMWH in combination with TA for VTE prophylaxis in TKA. Comprehensive data collection, including blood loss measurements and postoperative complications, increases the reliability of the findings. The results are consistent with the existing literature and support the study's validity. Limitations of our study include the retrospective design, which limits the ability to establish a causal relationship. Unbalanced sample sizes between groups and small overall sample sizes reduce statistical power. Differences in surgical techniques and implant types negatively affect the standardization of the results. The study was specific to a single center, which may limit generalizability. Multicenter prospective studies with larger sample sizes are required to confirm these findings.

## Conclusions

This study compared the efficacy and safety of the antifibrinolytic agent tranexamic acid in combination with acetylsalicylic acid and low-molecular-weight heparin in knee arthroplasty. The results show that acetylsalicylic acid and tranexamic acid provide lower total blood loss, less drainage volume, and lower blood transfusion requirement. These findings suggest that acetylsalicylic acid may be preferred as a safe and effective thromboprophylaxis agent. However, confirmation with larger sample sizes and prospective studies is required.
